# *In vitro* and *in vivo* anti-leukemic effects of cladoloside C_2_ are mediated by activation of Fas/ceramide synthase 6/p38 kinase/c-Jun NH_2_-terminal kinase/caspase-8

**DOI:** 10.18632/oncotarget.23069

**Published:** 2017-12-08

**Authors:** Seong-Hoon Yun, Eun-Hye Sim, Sang-Heum Han, Tae-Rang Kim, Mi-Ha Ju, Jin-Yeong Han, Jin-Sook Jeong, Sung-Hyun Kim, Alexandra S. Silchenko, Valentin A. Stonik, Joo-In Park

**Affiliations:** ^1^ Department of Biochemistry, Dong-A University College of Medicine, Busan, South Korea; ^2^ Department of Pathology, Dong-A University College of Medicine, Busan, South Korea; ^3^ Department of Laboratory Medicine, Dong-A University College of Medicine, Busan, South Korea; ^4^ Department of Internal Medicine, Dong-A University College of Medicine, Busan, South Korea; ^5^ G.B. Elyakov Pacific Institute of Bio-organic Chemistry, Far-Eastern Branch of the Russian Academy of Sciences, Vladivostok, Russia

**Keywords:** marine triterpene glycoside, ceramide synthase 6, JNK, caspase-8, anti-leukemic activity

## Abstract

We previously demonstrated that the quinovose-containing hexaoside stichoposide C (STC) is a more potent anti-leukemic agent than the glucose-containing stichoposide D (STD), and that these substances have different molecular mechanisms of action. In the present study, we investigated the novel marine triterpene glycoside cladoloside C_2_ from *Cladolabes schmeltzii*, which has the same carbohydrate moiety as STC. We assessed whether cladoloside C_2_ could induce apoptosis in K562 and HL-60 cells. We also evaluated whether it showed antitumor action in mouse leukemia xenograft models, and its molecular mechanisms of action. We investigated the molecular mechanism behind cladoloside C_2_-induced apoptosis of human leukemia cells, and examined the antitumor effect of cladoloside C_2_ in a HL-60 and K562 leukemia xenograft model.

Cladoloside C_2_ dose- and time-dependently induced apoptosis in the analyzed cells, and led to the activation of Fas/ceramide synthase 6 (CerS6)/p38 kinase/JNK/caspase-8. This cladoloside C_2_-induced apoptosis was partially blocked by specific inhibition by Fas, CerS6, and p38 siRNA transfection, and by specific inhibition of JNK by SP600125 or dominant negative-JNK transfection. Cladoloside C_2_ exerted antitumor activity through the activation of Fas/CerS6/p38 kinase/JNK/caspase-8 without showing any toxicity in xenograft mouse models. The antitumor effect of cladoloside C_2_ was reversed in CerS6 shRNA-silenced xenograft models. Our results suggest that cladoloside C2 has *in vitro* and *in vivo* anti-leukemic effects due to the activation of Fas/CerS6/p38 kinase/JNK/caspase-8 in lipid rafts. These findings support the therapeutic relevance of cladoloside C_2_ in the treatment of human leukemia.

## INTRODUCTION

Leukemia is a heterogeneous clonal disorder characterized by defects in cell differentiation and the death of hematopoietic progenitor cells. Despite major advances in drug development, leukemia treatment remains limited by resistance to chemotherapeutic agents in many patients [1, 2]. Thus, there remains a need for new therapeutic agents and strategies to improving the leukemia cure rate.

The tumor-suppressor lipid ceramide reportedly exhibits potent growth inhibition effects in a variety of cell types [3]. Ceramide can be generated by either ceramide synthases or sphingomyelinases [4, 5]. Sphingomyelinases (SMases) are categorized as acid, neutral, or alkaline [6, 7], according to the pH at which they show maximum activity [8]. Many anticancer agents increase ceramide levels to varying extents in different types of cancer cells [9]. Thus, the pharmacological modulation of sphingolipid metabolism to enhance ceramide in tumor cells represents a novel therapeutic approach.

**Figure 1:**
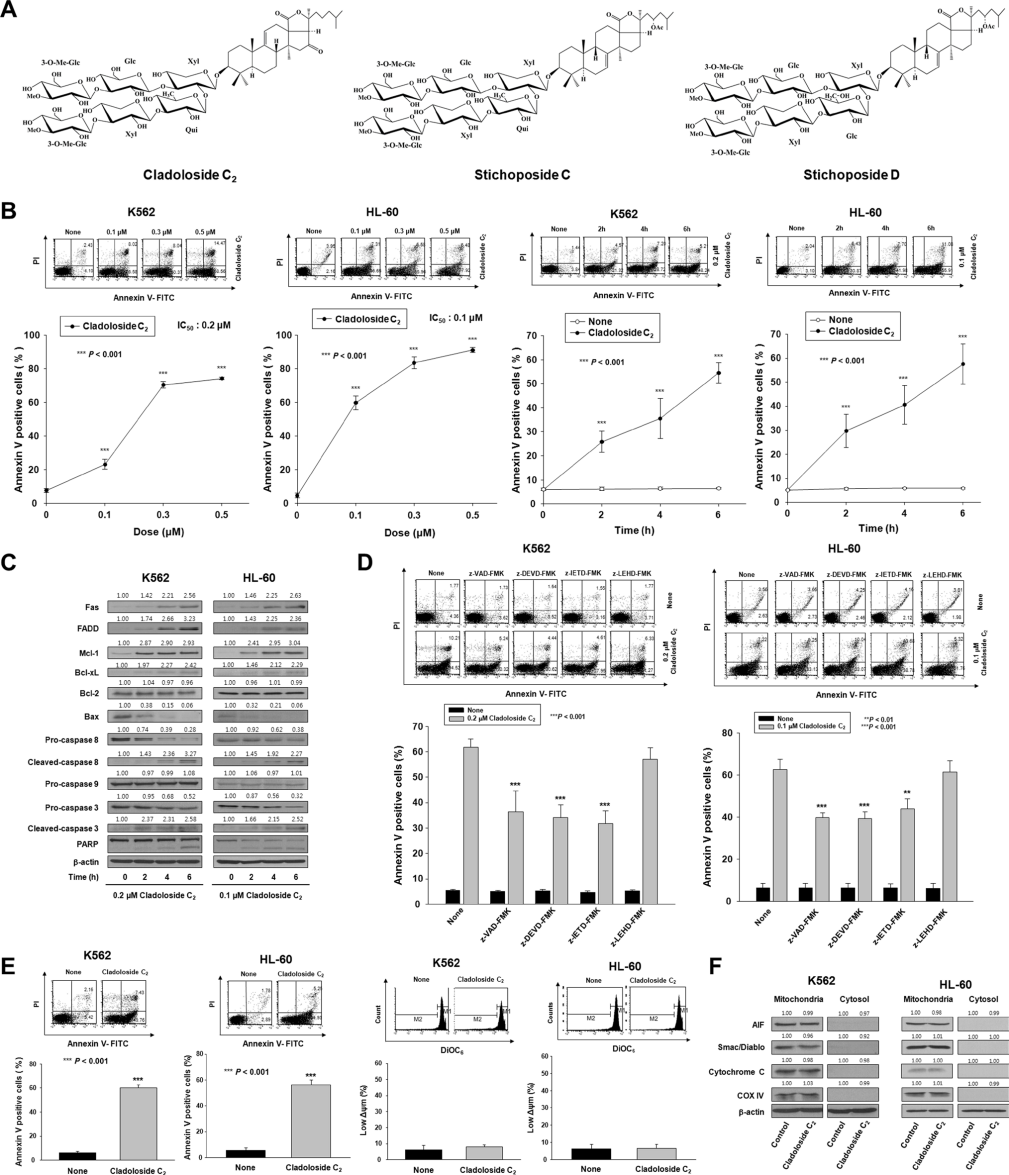
Cladoloside C_2_ induces apoptosis through extrinsic pathway activation in human leukemic cells (**A**) Structures of cladoloside C_2_ and stichoposides C and D. (**B**) Left panel: K562 and HL-60 cells were seeded, cultured for 4 h, and then treated for 6 h with various concentrations of cladoloside C_2_ (0, 0.1, 0.3, or 0.5 µM). Right panel: K562 and HL-60 cells were seeded, cultured for 4 h, and then treated for the indicated times with 0.2 or 0.1 µM cladoloside C_2_. The percentage of apoptotic cells was determined by annexin V-FITC/PI staining. Upper panels: Representative of three separate experiments. Lower panels: Mean ± SD of three independent experiments. ^***^P < 0.001 *vs.* control cells. (**C**) K562 and HL-60 cells were treated with 0.2 or 0.1 µM cladoloside C_2_ for the indicated times. Protein lysates were prepared and used for western blot analysis with the corresponding antibodies. β-actin was used as a loading control. The blot is representative of three separate experiments. (**D**) Functional involvement of caspases in cladoloside C_2_-induced apoptosis of K562 and HL-60 cells. Cells were pretreated for 1 h with the pan-caspase inhibitor Z-VAD-FMK (25 μM), the caspase-8 inhibitor Z-IETD-FMK (20 μM), the caspase-9 inhibitor Z-LEHD-FMK (20 μM), or the caspase-3 inhibitor Z-DEVD-FMK (50 μM), followed by treatment with 0.2 or 0.1 μM cladoloside C_2_ for 6 h. The extent of apoptosis was measured by flow cytometry after annexin V staining. These data represent the mean ± SD of three independent experiments. ^**^P < 0.01; ^***^P < 0.001 *vs.* cladoloside C_2_-treated cells. (**E**) Left panel: K562 and HL-60 cells were treated with 0.2 or 0.1 μM cladoloside C_2_ for 6 h. The extent of apoptosis was measured by flow cytometry after annexin V staining. These data represent the mean ± SD of three independent experiments. ^***^P < 0.001 *vs.* control cells. Right panel: K562 and HL-60 cells were treated with 0.2 or 0.1 mM cladoloside C_2_ for 4 h or 2 h. The cells were stained with DiOC_6_, and the reduction in Δφ_m_ was determined by monitoring the DiOC_6_ uptake using flow cytometry. Low Δφ_m_ values are expressed as the percentage of cells exhibiting diminished mitochondrial potential. The values obtained from the DiOC_6_ assays represent the mean ± SD of three independent experiments. (**F**) Western blot for the mitochondrial proteins AIF, Smac/DIABLO, cytochrome oxidase IV, and cytochrome c. Cytochrome oxidase IV (COX IV) was used as a mitochondrial marker. Protein lysates were prepared and subjected to western blot analysis using corresponding antibodies. β-actin was used as a loading control. The blot is representative of three separate experiments.

Marine triterpene glycosides exhibit a wide range of biological activities, including antitumor activity [10, 11]. We previously demonstrated that stichoposide C (STC) (Figure 1A)—a hexaoside containing quinovose as the second monosaccharide unit—induces apoptosis of leukemia cells by generating ceramide. The mechanisms for ceramide generation include activation of acid SMase after activating caspase-8, and the activation of neutral SMase resulting from glutathione depletion and increased ROS production [12]. Another study reported that stichoposide D (STD) (Figure 1A)—a STC structural analog that contains glucose instead of quinovose in its carbohydrate chains—induces leukemia cell apoptosis through the activation of ceramide synthase 6 (CerS6) [13]. These studies suggest that marine triterpene glycosides, particularly hexaosides containing a quinovose as the second monosaccharide, are strong candidates for anti-leukemic agents.

Cladoloside C_2_, a novel marine triterpene glycoside with a quinovose as the second monosaccharide (Figure 1A), has been isolated from the holothurian *Cladolabes schmeltzii* (Subfamily Cladolabinae, Family Sclerodactylidae, Order Dendrochirotida). In our present study, we investigated the molecular mechanism underlying the anti-leukemic potential of cladoloside C_2_ in K562 and HL-60 cells, and mouse leukemia xenograft models. Our data provide the first evidence that cladoloside C_2_ induces apoptosis of human leukemic cells through an extrinsic, but not an intrinsic pathway. We further demonstrated that the *in vitro* and *in vivo* anti-leukemic effects of cladoloside C_2_ occur through a mechanism involving the activation of Fas/CerS6/p38 kinase/c-Jun-NH_2_-terminal kinase (JNK)/caspase-8 in lipid rafts.

## RESULTS

### Cladoloside C_2_ induces apoptosis of leukemic cells through extrinsic pathway activation

To examine whether cladoloside C_2_ can induce apoptosis of K562 and HL-60 cells, K562 and HL-60 cells were treated with various cladoloside C_2_ concentrations for different time periods, and co-stained with PI and FITC-conjugated annexin V. Cladoloside C_2_ treatment dose- and time-dependently increased the proportions of apoptotic cells (Figure 1B). In contrast, the concentrations of cladoloside C_2_ that were used in this study (0.1–1.0 μM) did not increase apoptosis of normal human hematopoietic progenitor cells (CD34^+^ cells) compared to control, as further confirmed by annexin-V/PI staining (data not shown).

We further evaluated the cladoloside C_2_-induced apoptotic signaling in K562 and HL-60 cells, with particular focus on the caspase activation cascade. Cladoloside C_2_-induced caspase activation was suggested by cleavage of the caspase-3 substrate PARP, and was confirmed by the presence of cleaved caspase-3 and caspase-8 (Figure 1C). To investigate the functional involvement of caspases in cladoloside C_2_-induced apoptosis, we used the pan-caspase inhibitor (Z-VAD-FMK), and specific inhibitors of caspase-3 (Z-DEVD-FMK), caspase-8 (Z-IETD-FMK), and caspase-9 (Z-LEHD-FMK). Cladoloside C_2_-induced apoptosis was partially abolished by pretreatment with Z-VAD-FMK, Z-DEVD-FMK, or Z-IETD-FMK, but not Z-LEHD-FMK (Figure 1D). These data suggest that cladoloside C_2_-induced apoptosis in K562 and HL-60 cells is influenced by a caspase-dependent mechanism involving an extrinsic pathway.

To assess mitochondrial pathway activation by cladoloside C_2_ treatment, we measured the mitochondrial membrane potential (MMP) and examined mitochondrial protein expression in the cytosol using western blot analysis. Cladoloside C_2_-treated K562 and HL-60 cells showed no MMP loss (Figure 1E), as well as no cytoplasmic release of cytochrome c, Smac/DIABLO, or AIF (Figure 1F). These findings indicate that cladoloside C_2_ treatment of K562 and HL-60 cells activated extrinsic apoptotic pathways, but not intrinsic pathways. To explain this phenomenon, we further investigated how cladoloside C_2_ treatment affected the levels of the antiapoptotic proteins myeloid cell leukemia-1 (Mcl-1), B-cell lymphoma-2 (Bcl-2), and B-cell lymphoma extra large (Bcl-xL); and the proapoptotic protein Bcl-2-associated X protein (Bax). Interestingly, cladoloside C_2_ led to increased expressions of Mcl-1 and Bcl-xL, decreased expression of Bax, and no change in Bcl-2 expression (Figure 1C)_._ The decreased Bax expression and increased expressions of Mcl-1 and Bcl-xL may support mitochondrial preservation.

### Cladoloside C_2_ generates ceramide through activation of ceramide synthase 6 following Fas activation in human leukemic cells

**Figure 2:**
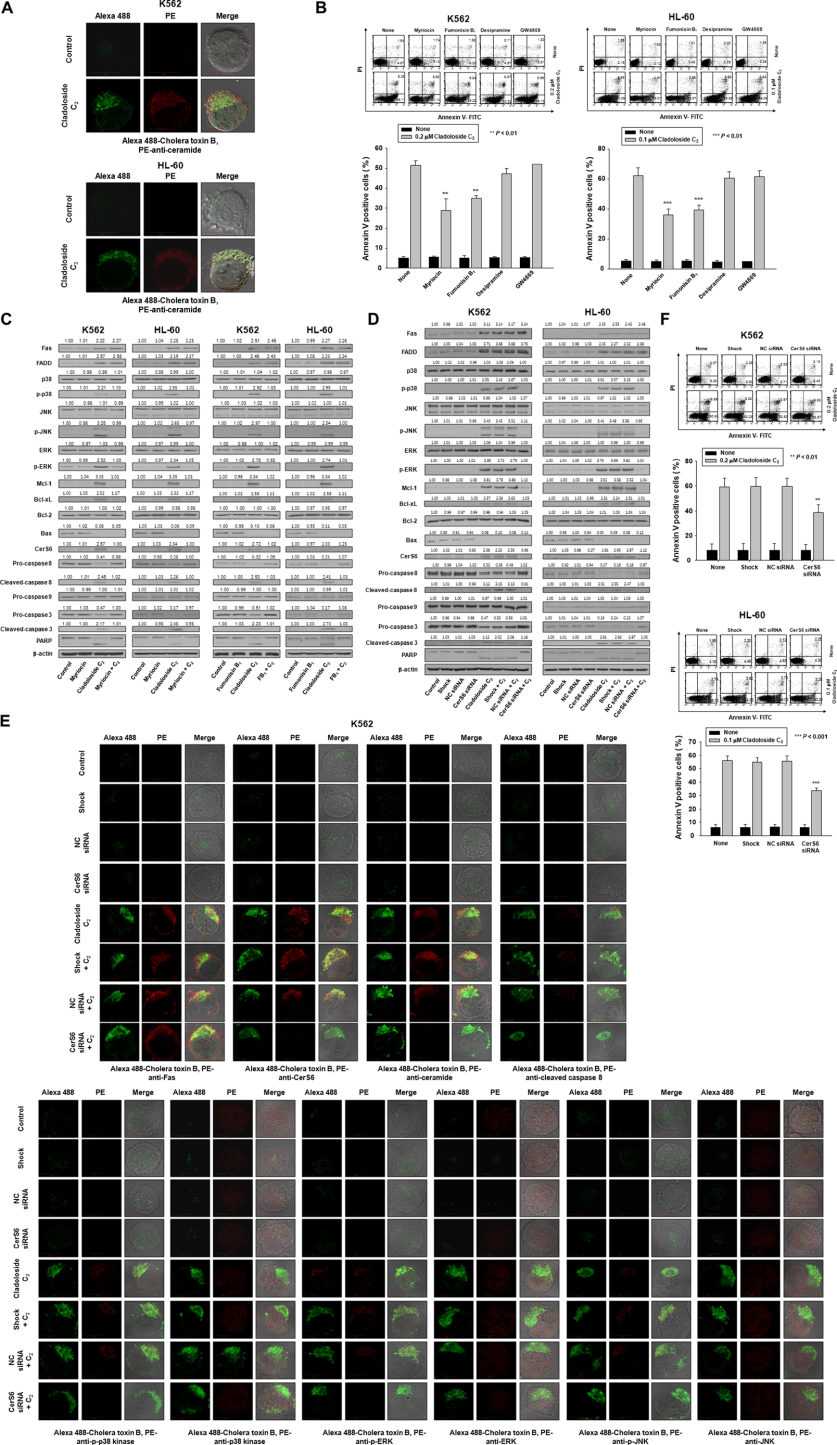
Cladoloside C_2_ induces apoptosis of K562 and HL-60 cells through the activation of ceramide synthase 6 (CerS6) (**A**) K562 and HL-60 cells treated with cladoloside C_2_ exhibited increased ceramide generation. (**B**) K562 and HL-60 cells (1 × 10^5^ cells/well) were incubated for 6 h with cladoloside C_2_ in the presence or absence of myriocin, fumonisin B_1_, desipramine, or GW4869. After treatment, the percentage of apoptotic cells was determined by annexin V-FITC/PI staining. Upper panel: Representative of three experiments in each cell line. Lower panel: Mean ± SD of three independent experiments. ^**^P < 0.01; ^***^P < 0.001 *vs.* cladoloside C_2_-treated cells. (**C**) K562 and HL-60 cells were incubated for 6 h with cladoloside C_2_ in the presence or absence of myriocin or fumonisin B_1_. Protein lysates were prepared and subjected to western blot analysis using corresponding antibodies. Western blots are each representative of three separate experiments. β-actin was used as a loading control. Densitometry results are expressed above the bands. (**D**–**F**) K562 and HL-60 cells were transiently transfected for 48 h by electroporation with CerS6 siRNA, nonspecific control (NC) siRNA, or no siRNA (shock). (D) Western blot analysis of protein lysates. (E) Transfected K562 cells were exposed to 0.2 μM cladoloside C_2_ for 2 h, and then fixed and permeabilized. Samples were then stained with PE-conjugated antibodies against Fas, CerS6, ceramide, cleaved caspase-8, p-p38 kinase, p38 kinase, p-ERK, ERK, p-JNK, or JNK, and with Alexa 488-labeled cholera toxin B antibody. The pictures are representative of three separate experiments. (F) Upper panel: The culture medium was changed, and K562 and HL-60 cells were incubated for 6 h with or without 0.2 or 0.1 μM cladoloside C_2_. The percentage of apoptotic cells was determined by annexin V-FITC/PI staining. Results are representative of three independent experiments in each cell line. Lower panel: Mean ± SD of three independent experiments. ^**^P < 0.01, ^***^P < 0.001, cells treated with cladoloside C_2_ alone versus cells transfected with CerS6 siRNA and treated with cladoloside C_2_.

We previously demonstrated that quinovose-containing STC induces apoptosis through ceramide generation via activation of acid and neutral SMase [12]. We expected that cladoloside C_2_ and STC would induce apoptosis through the same mechanism. Thus, we performed immunofluorescence staining, and found that cladoloside C_2_ increased ceramide generation (Figure 2A). To evaluate whether cladoloside C_2_-induced apoptosis was mediated by acid SMase, neutral SMase, or ceramide synthase, we incubated cells for 1 h with the acid SMase inhibitor desipramine, the neutral SMase inhibitor GW4869, the serine palmitoyl transferase inhibitor myriocin, or the ceramide synthase inhibitor fumonisin B_1_, followed by treatment with cladoloside C_2_. Cladoloside C_2_-induced apoptosis was partially blocked by pretreatment with myriocin or fumonisin B_1_, and was not blocked by pretreatment with desipramine or GW4869 (Figure 2B). Cladoloside C_2_-induced ceramide generation in K562 and HL-60 cells was also blocked by pretreatment with myriocin or fumonisin B_1_ (data not shown). We also examined the expressions of CerS4, CerS5, and CerS6 during cladoloside C_2_-induced apoptosis. Cladoloside C_2_ treatment markedly induced expression of CerS6, but not CerS4 or CerS5 (Supplementary Figure 1A, 1B). Thus, we focused on the role of CerS6 in cladoloside C_2_-mediated cell death. Western blot analysis and immunofluorescence staining revealed that cladoloside C_2_ treatment increased CerS6 expression in K562 and HL-60 cells (Figure 2C).

To verify the essential role of CerS6 activation in cladoloside C_2_-mediated apoptosis, we transfected K562 and HL-60 cells with siRNA against CerS6 or nonspecific control siRNA. Western blot analysis and immunofluorescence staining confirmed CerS6 knockdown (Figure 2D, 2E). The extent of apoptosis was monitored in cladoloside C_2_-treated transfected cells. CerS6 knockdown by siRNA partially protected cells from cladoloside C_2_-induced apoptosis (Figure 2F). We observed similar results in CerS6 shRNA-silenced K562 and HL-60 cells (Supplementary Figure 2A–2C).

To establish the sequence of events accompanying cladoloside C_2_-induced cell death, we monitored cladoloside C_2_-induced activation of Fas, caspase-8, and caspase-3 in CerS6 siRNA-transfected K562 and HL-60 cells. CerS6 siRNA transfection reversed the activation of caspase-8 and caspase-3, but not Fas (Figure 2D). These data suggest that CerS6 activation occurred downstream of Fas activation and upstream of caspase-8 and caspase-3.

### Fas activation is involved in cladoloside C_2_-induced CerS6 activation and apoptosis

**Figure 3:**
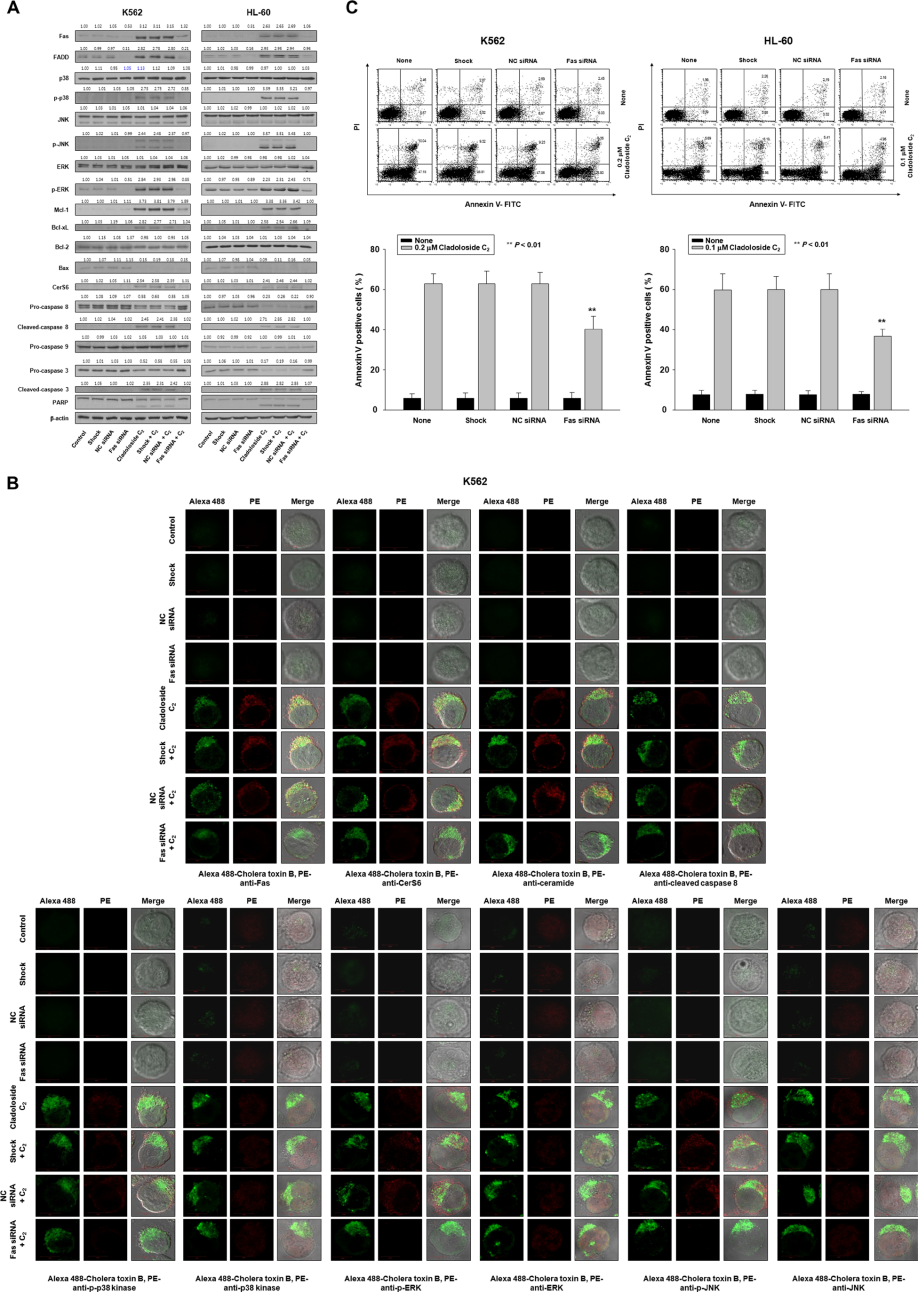
Fas knockdown inhibits cladoloside C_2_-induced apoptosis in K562 and HL-60 cells K562 and HL-60 cells were transiently transfected for 48 h by electroporation with Fas siRNA, nonspecific control (NC) siRNA, or no siRNA (shock). (**A**) Western blot analysis of protein lysates. (**B**) Transfected K562 cells were treated with cladoloside C_2_ for 2 h, and then fixed and permeabilized. The samples were then stained with PE-conjugated antibodies against Fas, CerS6, ceramide, cleaved caspase-8, p-p38 kinase, p38 kinase, p-ERK, ERK, p-JNK, or JNK, as well as Alexa 488-labeled cholera toxin B antibody. The pictures are representative of three separate experiments. (**C**) The culture medium was changed, and cells were incubated for 6 h with or without cladoloside C_2_. The percentage of apoptotic cells was determined by annexin V-FITC/PI staining. Upper panels: Representative of three independent experiments in each cell line. Lower panels: Mean ± SD of three independent experiments. ^**^P < 0.01, cells treated with cladoloside C_2_ versus cells transfected with Fas siRNA and treated with cladoloside C_2_.

Since cladoloside C_2_ treatment activated Fas (Figure 1C), we evaluated the functional significance of this activation by performing Fas knockdown with Fas siRNA in K562 and HL-60 cells. Western blot analysis and immunofluorescence staining confirmed Fas knockdown (Figure 3A, 3B), and the extent of apoptosis was monitored in cladoloside C_2_-treated transfected cells. Fas knockdown partially protected cells from cladoloside C_2_-induced apoptosis (Figure 3C), and reduced cladoloside C_2_-induced CerS6 activation and ceramide generation (Figure 3A, 3B). Additionally, Fas siRNA transfection inhibited cladoloside C_2_-induced activation of caspase-8 and caspase-3 (Figure 3A).

### Activation of p38 kinase and JNK occurs downstream of Fas and CerS6 activation, and may contribute to cladoloside C_2_-induced apoptosis in human leukemic cells

**Figure 4:**
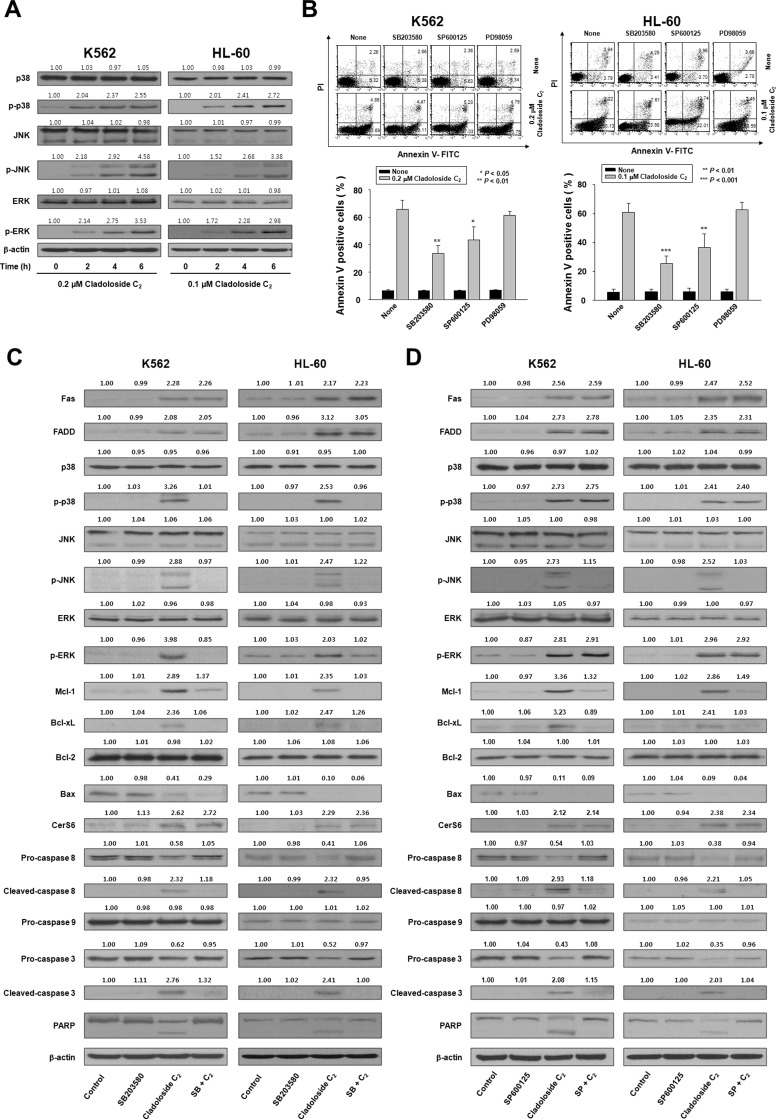
Cladoloside C_2_ induces apoptosis of K562 and HL-60 cells through activation of p38 kinase and JNK (**A**) K562 and HL-60 cells were treated with cladoloside C_2_ for the indicated times. Protein lysates were prepared and subjected to western blot analysis. β-actin was used as a loading control. The blot is representative of three separate experiments. (**B**) K562 and HL-60 cells (1 × 10^5^ cells/well) were pretreated with the p38 kinase inhibitor SB203580, the JNK inhibitor SP600125, or the ERK inhibitor PD98059, followed by 6 h of treatment with 0.2 or 0.1 μM cladoloside C_2_. After treatment for the indicated times, the percentage of apoptotic cells was determined by annexin V-FITC/PI staining. Upper panel: Representative of three independent experiments. Lower panel: Mean ± SD of three independent experiments. ^*^P < 0.05; ^**^P < 0.01; ^***^P < 0.001, versus cells treated with cladoloside C_2_ in the absence of SB203580 or SP600125. (**C** and **D**) K562 and HL-60 cells (1 × 10^5^ cells/well) were pretreated with the p38 kinase inhibitor SB203580 (C) or the JNK inhibitor SP600125 (D), followed by treatment with 0.2 or 0.1 μM cladoloside C_2_ for 6 h. Protein lysates were prepared and subjected to western blot analysis using corresponding antibodies. Western blots are each representative of three separate experiments. β-actin was used as a loading control. Densitometry results are expressed above the bands.

Ceramide activates multiple signaling pathways, including those involving mitogen-activated protein kinases (MAPKs) [14–18]. MAPKs—such as the extracellular signal regulating kinase (ERK), p38 kinase, and JNK—are centrally involved in stress-induced cell death, and in apoptotic signaling of ceramide. We explored the involvement of ERK, p38 kinase, and JNK in cladoloside C_2_-induced apoptosis by treating K562 and HL-60 cells with cladoloside C_2_ for various time periods, and then measuring MAPK protein levels by western blot analysis. Cladoloside C_2_ time-dependently activated all MAPKs (Figure 4A). To ascertain the roles of activated ERK, p38 kinase, and JNK in cladoloside C_2_-induced cell death, we used specific inhibitors of ERK (PD98059), p38 kinase (SB203580), and JNK (SP600125) and measured the extent of apoptosis after 6 h of cladoloside C_2_ treatment. Apoptosis was significantly reduced by inhibition of p38 kinase and JNK, but not ERK (Figure 4B). Inhibition of p38 kinase and JNK also significantly inhibited cladoloside C_2_-induced caspase-8 activation, but not Fas activation, CerS6 activation, or ceramide generation (Figure 4C, 4D).

**Figure 5:**
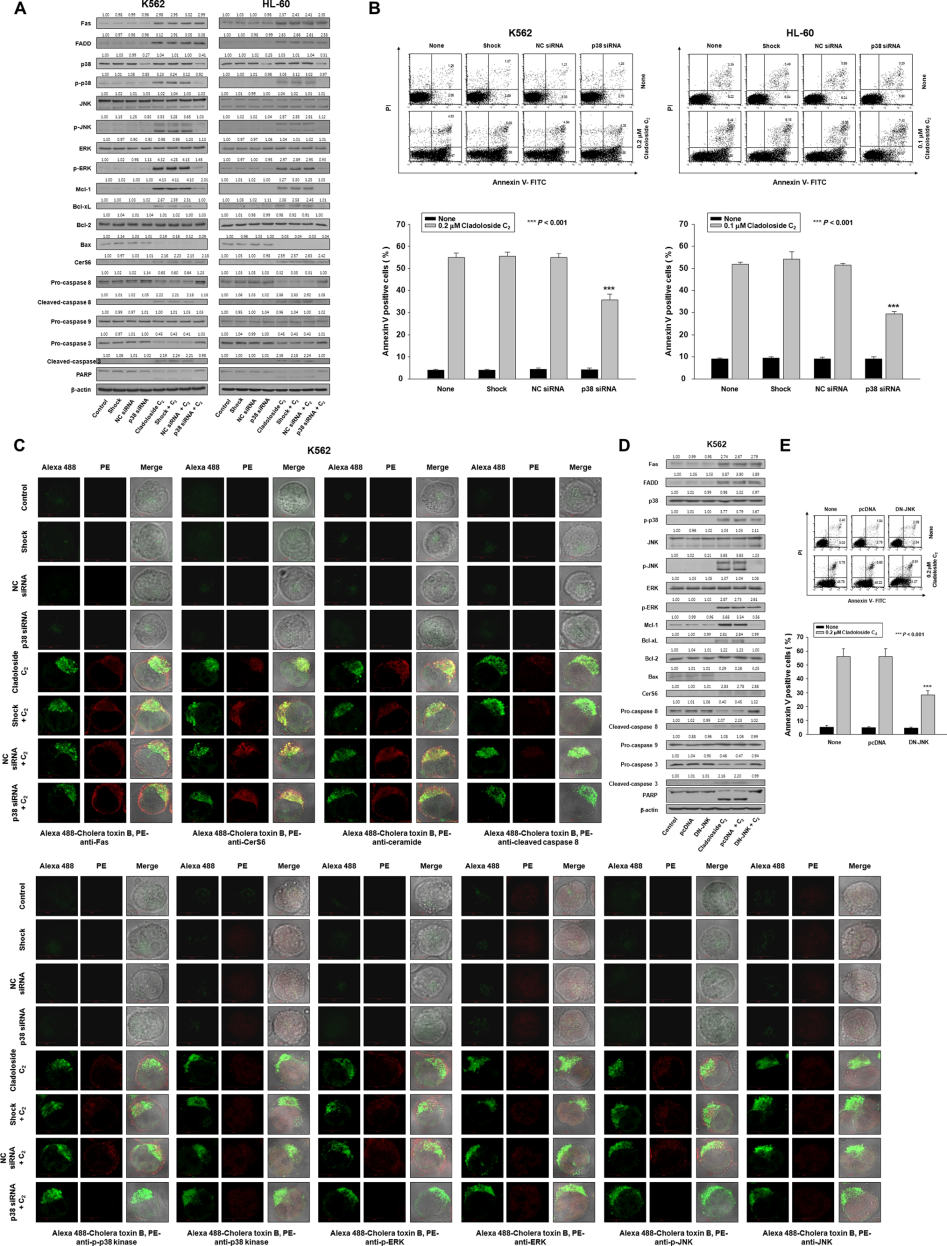
p38 kinase knockdown and DN-JNK transfection inhibit cladoloside C_2_-induced apoptosis in K562 and HL-60 cells (**A**–**C**) K562 and HL-60 cells were transiently transfected by electroporation for 48 h with p38 siRNA, nonspecific control (NC) siRNA, or no siRNA (shock). (A) Transfected K562 and HL-60 cells were incubated for 6 h with or without cladoloside C_2_. Protein lysates were prepared and subjected to western blot analysis. (B) The culture medium was changed, and cells were incubated for 6 h with or without cladoloside C_2_. The percentage of apoptotic cells was determined by annexin V-FITC/PI staining. Upper panel: Representative of three independent experiments in each cell line. Lower panel: Mean ± SD of three independent experiments. ^***^P < 0.001, cells treated with cladoloside C_2_ versus cells transfected with p38 kinase siRNA and treated with cladoloside C_2_. (C) Transfected K562 cells were treated with cladoloside C_2_ for 2 h, and then fixed and permeabilized. The samples were then stained with PE-conjugated antibodies against Fas, CerS6, ceramide, cleaved caspase-8, p-p38 kinase, p38 kinase, p-ERK, ERK, p-JNK, or JNK, as well as Alexa 488-labeled cholera toxin B antibody. The pictures are representative of three separate experiments. (**D** and **E**) K562 cells were transiently transfected by electroporation for 48 h with pcDNA or DN-JNK plasmid. (D) Transfected K562 cells were incubated for 6 h with or without cladoloside C_2_. Protein lysates were prepared and subjected to western blot analysis. (E) The culture medium was changed, and cells were incubated for 6 h with or without cladoloside C_2_. The percentage of apoptotic cells was determined by annexin V-FITC/PI staining. Upper panel: Representative of three independent experiments in K562 cells. Lower panel: Mean ± SD of three independent experiments. ^***^P < 0.001, cells treated with cladoloside C_2_ versus cells transfected with DN-JNK and treated with cladoloside C_2_.

To further confirm the crucial role of p38 kinase in cladoloside C_2_-induced apoptosis, we transfected K562 and HL-60 cells with p38 kinase siRNA. Western blot analysis confirmed p38 kinase knockdown (Figure 5A), and the extent of apoptosis was monitored in cladoloside C_2_-treated transfected cells. Knockdown of p38 kinase partially protected cells from cladoloside C_2_-induced apoptosis (Figure 5B), but did not reduce cladoloside C_2_-induced Fas and CerS6 activation (Figure 5A, 5C). These siRNA experiments also revealed inhibition of caspase-8 and caspase-3 activation (Figure 5A).

To ascertain whether JNK activation was necessary for cladoloside C_2_-mediated apoptosis, K562 cells were transiently transfected with a dominant-negative JNK expression vector (DN-JNK) expression vector or empty vector. Western blot analysis confirmed JNK inhibition (Figure 5D), and the extent of apoptosis was monitored in cladoloside C_2_-treated transfected cells. JNK inhibition partially protected cells from cladoloside C_2_-induced apoptosis (Figure 5E), but did not reduce cladoloside C_2_-induced activation of Fas, CerS6, or p38 kinase (Figure 5D). DN-JNK transfection also inhibited activation of caspase-8 and caspase-3 (Figure 5D).

### Clustering of Fas and its downstream signaling molecules in lipid rafts during cladoloside C_2_-induced apoptosis

**Figure 6:**
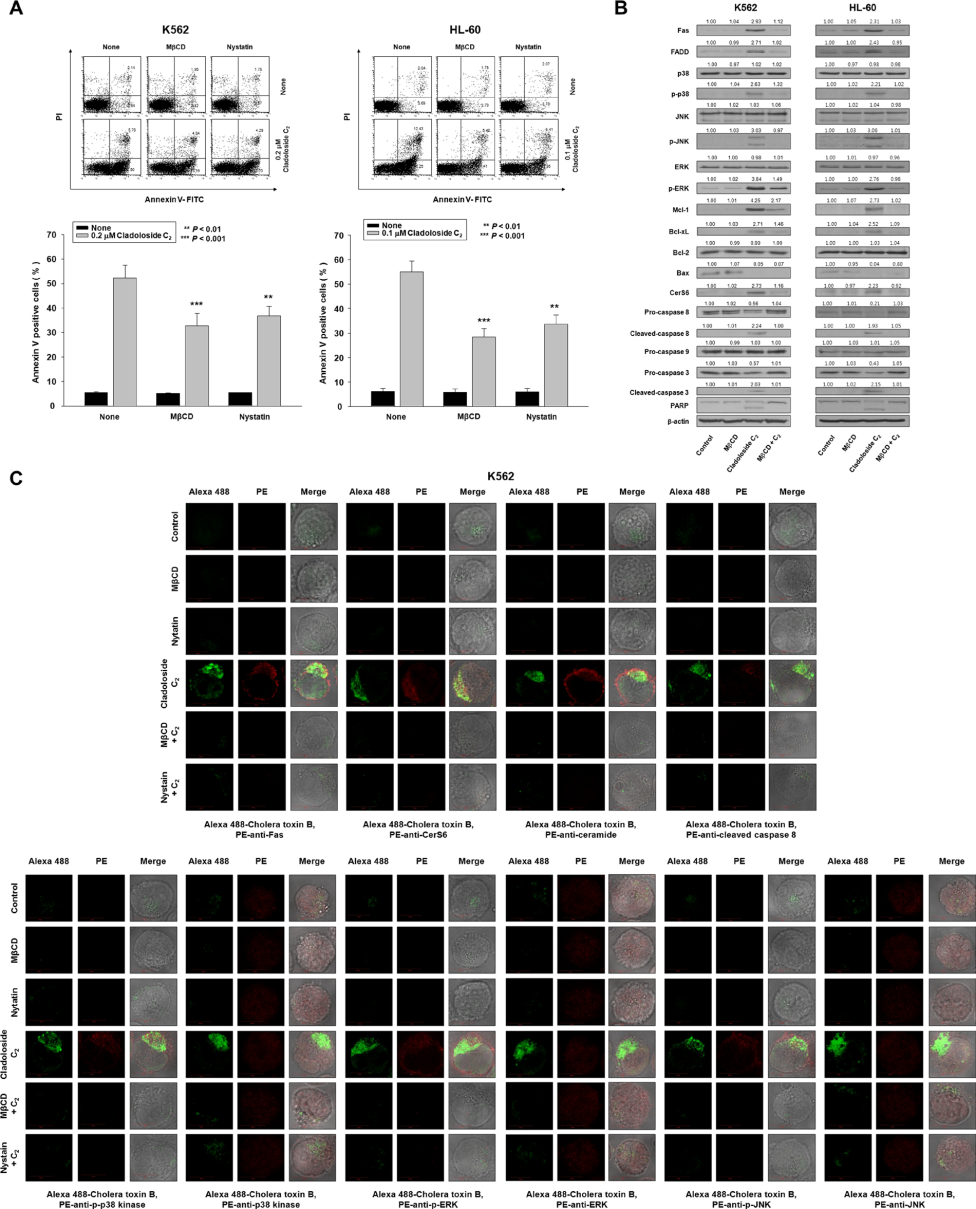
Clustering of Fas and its downstream molecules in lipid rafts during cladoloside C_2_-induced apoptosis of K562 and HL-60 cells (**A**) K562 and HL-60 cells were pretreated for 1 h with MβCD (20 μg/mL) and nystatin (20 μg/mL), and then cultured for 6 h in medium containing 0.2 or 0.1 μM cladoloside C_2_. After treatment for the indicated times, the percentage of apoptotic cells was determined by annexin V-FITC/PI staining. Upper panel: Representative of three independent experiments. Lower panel: Mean ± SD of three independent experiments. ^**^P < 0.01; ^***^P < 0.001 versus cladoloside C_2_-treated cells. (**B**) Whole cell lysates were prepared from K562 and HL-60 cells incubated for 6 h with cladoloside C_2_ in the presence or absence of MβCD, and were subjected to western blot analysis. (**C**) K562 cells were treated for 2 h with cladoloside C_2_ in the presence or absence of MβCD or nystatin, and then fixed and permeabilized. These samples were then stained with PE-conjugated antibodies against Fas, CerS6, ceramide, cleaved caspase-8, p-p38 kinase, p38 kinase, p-ERK, ERK, p-JNK, or JNK, as well as Alexa 488-labeled cholera toxin B antibody. The pictures are representative of three separate experiments.

The above-described observations suggest that cladoloside C_2_ activated Fas and CerS6, leading to ceramide generation, followed by the activation of p38 kinase, JNK, and caspase-8. We next examined the clustering of these signaling molecules (Fas, CerS6, ceramide, p38 kinase, JNK, and active caspase-8) in lipid rafts during cladoloside C_2_-induced apoptosis. K562 and HL-60 cells were pretreated with the cholesterol-depleting agent methyl-β-cyclodextrin (MβCD) and nystatin for 1 h, followed by cladoloside C_2_ treatment. Then we assessed the extent of apoptosis and the activation of Fas, caspase-8, caspase-3, and CerS6. Incubation of K562 and HL-60 cells with MβCD and nystatin inhibited cladoloside C_2_-induced apoptosis, as well as the activation of Fas, caspase-8, caspase-3, and CerS6 (Figure 6A–6C).

The cholera toxin (CTx) B subunit predominantly localizes in lipid rafts. We used the lipid raft marker Alexa 488-labeled CTx B, and found that cladoloside C_2_ promoted the co-aggregation of Fas/CD95, CerS6, ceramide, p-p38 kinase, p-JNK, and active caspase-8, with lipid rafts in K562 and HL-60 cells (Figure 6C). Furthermore, incubation of K562 and HL-60 cells with MβCD inhibited cladoloside C_2_-induced activation of caspase-8 and caspase-3 (Figure 6B).

### Cladoloside C_2_ induces antitumor activity through the activation of Fas, CerS6, p38 kinase, and JNK in K562 and HL-60 xenograft mouse tumor models

**Figure 7:**
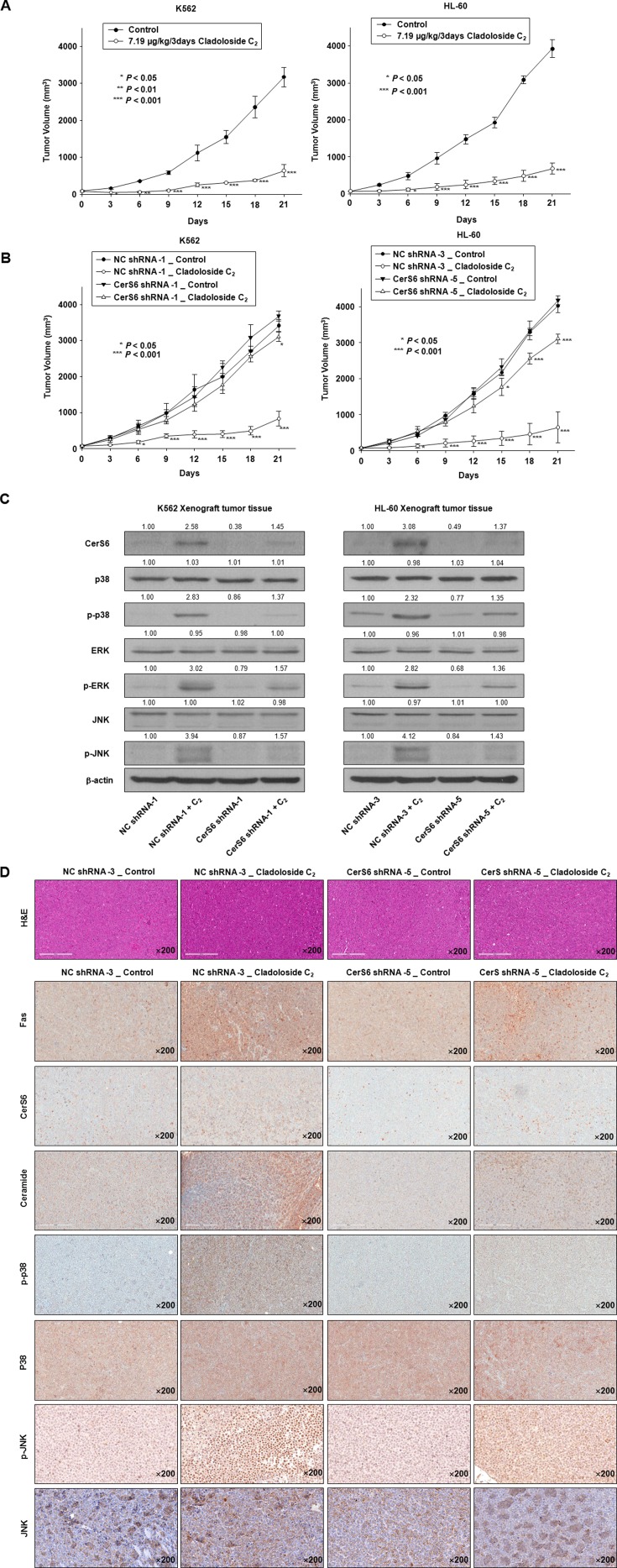
Cladoloside C_2_ inhibits the growth of K562 and HL-60 xenograft tumors and induces apoptosis through Fas/CerS6/p38 kinase/JNK activation *in vivo* (**A**) K562 (1.5 × 10^7^ cells/100 μL) and HL-60 cells (2 × 10^7^ cells/100 μL) were subcutaneously injected into Balb/c nude mice. After the cells formed palpable tumors, the mice were randomized into two groups (*n* = 5 mice/group) and treated with vehicle control or cladoloside C_2_ (7.19 μg/kg). Tumor size was measured daily using a caliper: calculated volume = shortest diameter^2^ × longest diameter/2. ^*^P < 0.05, ^**^P < 0.01, and ^***^P < 0.001, versus respective controls. (**B**) Left panel: NC-shRNA-transfected and CerS shRNA-transfected stable K562 (1.5 × 10^7^ cells/100 mL) were subcutaneously injected into Balb/c nude mice. Right panel: NC-shRNA-transfected and CerS shRNA-transfected stable HL-60 (2 × 10^7^ cells/100 mL) were subcutaneously injected into Balb/c nude mice. After the cells formed palpable tumors, the mice were randomized into two groups (*n* = 5 mice/group), and treated with vehicle control or cladoloside C_2_ (7.19 μg/kg). Tumor size was measured daily using a caliper. ^*^P < 0.05, ^**^P < 0.01, and ^***^P < 0.001, versus respective controls. (**C** and **D**) Tumor tissues obtained from the above-described experiment on Day 14 were subjected to western blot analysis (C) and immunohistochemistry (D) using antibodies against Fas, CerS6, ceramide, p-p38, p38 kinase, p-JNK, or JNK. The sections were lightly counterstained with hematoxylin, and photographed with a ScanScope. K562 and HL-60 leukemia xenografts from cladoloside C_2_-treated mice exhibited apoptosis and extensive necrosis (200×).

Finally, we observed that cladoloside C_2_ significantly inhibited tumor growth in both HL-60 and K562 mouse xenograft models (Figure 7A). Tumors from control mice displayed the typical histologic appearance of leukemic cells. After 21 d, tumors from cladoloside C_2_-treated mice showed mean volumes over 75% smaller than the volumes of tumors in vehicle-treated K562 and HL-60 xenograft mice (control group: 3174.18 ± 262.39 mm^3^, 3922.49 ± 241.42 mm^3^; cladoloside C_2_ group: 640.27 ± 159.74 mm^3^, 685.51 ± 154.62 mm^3^). We used stable CerS6 shRNA-silenced K562 and HL-60 xenograft models to investigate the involvement of CerS6 in the *in vivo* antitumor activity of cladoloside C_2_. In parallel, CerS6-silenced cells and nonspecific control (NC) cells were subcutaneously inoculated into 6-week-old nude mice. Next, cladoloside C_2_ or vehicle was injected into each mouse. The anti-tumor effect of cladoloside C_2_ was significantly inhibited in CerS6 shRNA-silenced K562 and HL-60 xenograft models (Figure 7B), with 75.6% and 84.1% inhibition of tumor growth in NC-shRNA-1 and NC-shRNA-3 xenograft models, respectively, vs. 15.6% and 25.6% inhibition of tumor growth by cladoloside C_2_ in CerS6-shRNA-1 and CerS6-shRNA-5 xenograft models, respectively. As expected, western blot analysis of tumors from vehicle-treated NC-shRNA-1 and NC-shRNA-3 control mice revealed weak expressions of Fas, CerS6, p-p38, and p-JNK (Figure 7C), and we observed weak immunostaining for Fas, CerS6, ceramide, p-p38, and p-JNK (Figure 7D). In contrast, western blot and immunohistochemical analysis of tumors from cladoloside C_2_-treated NC-shRNA-1 and NC-shRNA-3 shRNA mice revealed up-regulation of Fas, CerS6, ceramide, p-p38, and p-JNK (Figure 7C, 7D). The expression and staining of p38 kinase and JNK did not differ between control and cladoloside C_2_-treated tumors (Figure 7C, 7D). These data were consistent with our *in vitro* findings.

## DISCUSSION

Marine triterpene glycosides represent promising candidate anticancer agents. However, their detailed molecular mechanisms have not been clearly defined. We previously reported that triterpene glycosides from *Thelenota anax* induce leukemic cell apoptosis through ceramide generation. However, molecular mechanisms and potencies differ among structurally different triterpene glycosides, and a previous study suggests that STC has more potent anti-leukemia cell activity than STD. Here we investigated the novel nonsulfated triterpene glycoside cladoloside C_2_, which contains the same carbohydrate chain as STC but with a different aglycone moiety. Our present results indicated that cladoloside C_2_ is more potent than STC. Moreover, unlike STC, cladoloside C_2_ apparently activates extrinsic apoptosis pathways, but not intrinsic pathways.

Interestingly, cladoloside C_2_ did not seem to affect mitochondria, as we observed increased expressions of Mcl-1 and Bcl-xL and decreased Bax expression. Many cancer cells are apt to be resistant to chemotherapeutic agents due to overexpression of prosurvival factors, such as Mcl-1, Bcl-2, and Bcl-xL. Therefore, these proteins are important targets for the development of new anti-cancer agents [19–21]. Since cladoloside C_2_ induces apoptosis despite enhancing the expressions of Mcl-1 and Bcl-xL, it may be useful as an anti-leukemic agent in leukemic cells that overexpress Mcl-1 and Bcl-xL. In particular, Mcl-1 overexpression reportedly leads to etoposide resistance [22], and we observed that cladoloside C_2_ sensitized K562 cells to etoposide and Ara-C (Supplementary Figure 3). Further studies are required to confirm this sensitizing effect in various leukemic cells that are resistant to chemotherapeutic agents.

Since cladoloside C_2_ and STC share the same carbohydrate chain structure, we expected that cladoloside C_2_, like STC, would induce apoptosis through the activation of acid and neutral SMases. Unexpectedly, we found that cladoloside C_2_ induced apoptosis through CerS6 activation, following to Fas activation, and the subsequent activation of p38 kinase/JNK/caspase-8. Similar to the mechanism of STD, Fas activation by cladoloside C_2_ was not reversed by transfection with CerS6 siRNA. However, CerS6 activation and ceramide generation were reversed by Fas siRNA transfection.

In ceramide-induced apoptosis, MAPKs are important signaling molecules. Several previous studies have demonstrated that ceramide causes ERK dephosphorylation, and induces p38 kinase and JNK phosphorylation [14–18]. Ceramide-activated p38 kinase and JNK generally contribute to the induction of cell apoptosis through mitochondrial damage and caspase activation. Our present results demonstrated that cladoloside C_2_-generated ceramide activated ERK, p38 kinase, and JNK, but did not induce mitochondrial damage. Since JNK and p38 kinase signaling target the anti-apoptotic Mcl-1 and Bcl-2 proteins [23–25], we examined how p38 kinase and JNK inhibition (by chemical inhibitors or p38 kinase siRNA or DN-JNK) influenced Mcl-1 and Bcl-2 expressions, caspase-8 activation, and apoptosis. Interestingly, inhibition of p38 kinase by SB203580 and of JNK by SP600125 led to reduced Mcl-1 expression, and reversed capase-8 activation and apoptosis, but did not change Bcl-2 expression. Similar results were obtained from silencing p38 kinase using p38 kinase siRNA, and inhibiting JNK via DN-JNK transfection. Thus, it appears that the activities of p38 kinase and JNK lead to enhanced Mcl-1 expression and are involved in cladoloside C_2_-induced apoptosis.

Lipid rafts play crucial roles in the Fas receptor death pathway [26, 27]. Based on the recently demonstrated role of plasma membrane lipid rafts in STD-induced apoptosis [13], here we wed MβCD and nystatin to investigate the roles of the Fas death receptor pathway and ceramide-enriched membrane domains in cladoloside C_2_-induced cell death. Cladoloside C_2_ induced Fas clustering in lipid rafts. We also detected co-localization of the Fas downstream signaling molecules CerS6, ceramide, p38 kinase, JNK, and caspase-8 in lipid rafts upon cladoloside C_2_ stimulation. MβCD blocked the cladoloside C_2_-induced clustering of Fas and the downstream signaling molecules, we well as apoptosis. These results suggest that the clustering of Fas and downstream signaling molecules in lipid rafts was essential for cladoloside C_2_-induced apoptosis.

Compared to controls, cladoloside C_2_ treatment significantly inhibited tumor growth in mouse HL-60 and K562 leukemic xenograft models. Cladoloside C_2_ treatment also led to up-regulation of Fas, CerS6, and ceramide and activation of p38 kinase, JNK, and caspase-8. The anti-tumor effect of cladoloside C_2_ was significantly prevented in CerS6 shRNA-silenced xenograft models. These results are consistent with the *in vitro* data. Moreover, cladoloside C_2_ showed a lack of toxicity towards normal hematopoietic progenitor cells and in mice, supporting it as a promising potential candidate for therapeutic use.

Future studies are needed to explore the antitumor activity of cladoloside C_2_ in other types of leukemia, including chemotherapy-resistant leukemia cells, and in other types of cancer. There also remains a need to investigate other molecular mechanisms that may be involved in cladoloside C_2_-induced apoptosis.

**Figure 8:**
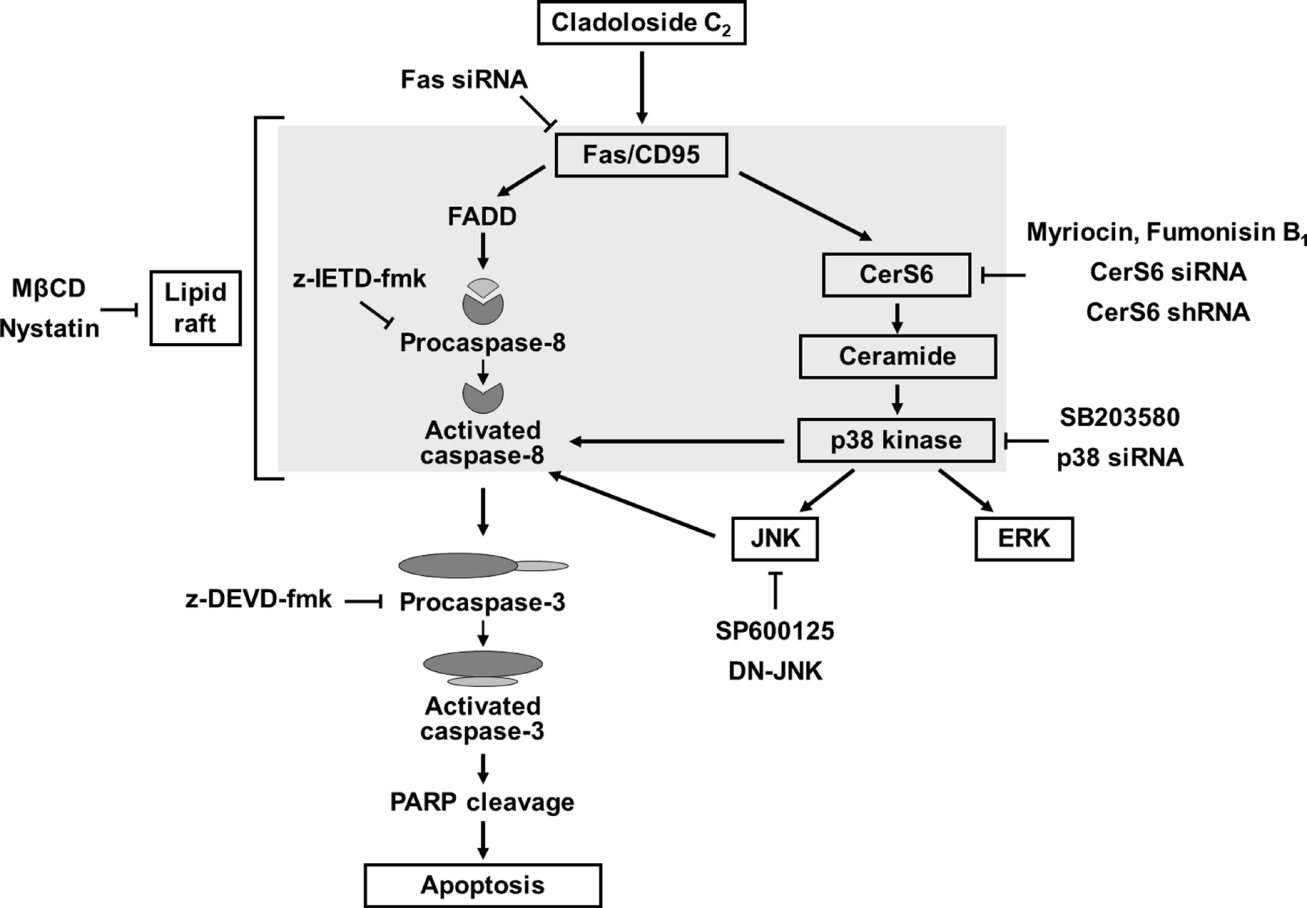
Hypothetical molecular mechanisms of cladoloside C_2_-induced apoptosis in human leukemia cells

In summary, this study provides the first evidence that cladoloside C_2_ induces apoptosis of human leukemic cells via activation of Fas/CerS6/p38 kinase/JNK/caspase-8 in lipid rafts (Figure 8). Cladoloside C_2_ also exhibited *in vivo* antitumor activity through the activation of Fas/CerS6/p38 kinase/JNK/caspase-8. Our results suggest that cladoloside C_2_ may be a useful candidate for the treatment of human leukemia overexpressing Mcl-1 and Bcl-xL.

## MATERIALS AND METHODS

### Cell preparations

The human leukemic cell lines K562 and HL-60 were obtained from the Korean Cell Line Bank (Seoul National University, Seoul, Korea), and cultured in RPMI1640 medium supplemented with 10% fetal bovine serum (FBS), 100 U/mL penicillin, and 100 μg/mL streptomycin. Human hematopoietic progenitor CD34^+^ cells were purchased from STEM CELL Technologies (Vancouver, BC), and cultured in Hematopoietic Progenitor Expansion Medium DXF with cytokine mix E (PromoCell, Heidelberg, Germany).

### Reagents

Cladoloside C_2_ was isolated and purified following the procedure published by Silchenko *et al.* [28], and was dissolved in sterilized distilled water. Annexin V was obtained from BD Biosciences Clontech (Palo Alto, CA, USA). Anti-Fas, anti-procaspase 8, anti-procaspase-3, anti-procaspase 9, and anti-cytochrome c antibodies were purchased from Santa Cruz Biotechnology (Santa Cruz, CA, USA). Antibodies against poly (ADP-ribose) polymerase (PARP), JNK, and p-JNK were purchased from Cell Signaling Technology (Beverly, MA, USA). The anti-β-actin antibody was obtained from Sigma (St. Louis, MO, USA). Unless otherwise stated, all other chemicals were purchased from Sigma.

### Apoptosis analysis

The extent of apoptosis was evaluated using annexin V-FITC and flow cytometry as previously described [29].

### Measurement of MMP

Variations in MMP (Δφ_m_) were examined using DiOC_6_ (Molecular Probes, Eugene, OR) as previously described [29].

### Separation of the cytosolic and mitochondrial proteins

Cytosolic and mitochondrial proteins were extracted from cells treated with sterilized water or with cladoloside C_2_ for the indicated times, and were separated as previously described [30, 31].

### Western blot analysis

Cell lysis and western blot analysis were performed as described previously [29], using 30 μg protein for immunoblotting. β-actin was used as the loading control.

### Immunofluorescence staining

Cells were fixed and permeabilized with 1% formaldehyde/methanol in PBS for 10 min at room temperature. Next, the cells were washed, and a series of antibodies was used as indicated, followed by staining with FITC- or PE-conjugated goat anti-mouse and anti-rabbit IgG (Calbiochem, San Diego, CA). The samples were then mounted using glycerol, and analyzed by confocal microscope (Carl Zeiss LSM 510; Carl Zeiss, Thornwood, NY) with a 40× C-Apochromat objective. Negative control staining was performed using only secondary antibodies.

### siRNA transfection

We purchased pre-designed siRNA targeted to human CerS6-1 mRNA (catalog number SI02758245; ID 253782), and AllStars negative control siRNA (catalog number 1027280) from Qiagen (Hilden, Germany). The siRNA sequence used for targeted silencing of CerS6 has been previously described [13]. Fas siRNA was obtained from Santa Cruz Biotechnology, and p38 kinase siRNA from Dharmacon (L-003512-00-0005; Thermo Scientific, Chicago, IL, USA), and their sequences have been previously published [13].

Cells were resuspended in PBS at 1.3 × 10^7^ cells/0.5 mL, and then mixed with 200 nM anti-CerS6 siRNA, anti-p38 kinase siRNA, anti-Fas siRNA, or non-silencing siRNA. This mixture was added to an electroporation cuvette with a 0.4-cm electrode gap, for transfection at 300 V and 950 μF in a Gene Pulser Xcell Electroporation System (Bio-Rad, Richmond, CA, USA). After electroporation, the cells were cultured for 48 h in RPMI1640 supplemented with 10% FBS, then treated with sterilized water or cladoloside C_2_ for the indicated times. These cells were then analyzed using annexin-V staining, immunofluorescence, and western blot.

### Generation of CerS6-silenced K562 and HL-60 cell lines

We obtained an shRNA construct containing CerS6 shRNA (MISSION^®^ shRNA plasmid DNA; CerS6-pLKO.1-puro) and the non-targeting control construct NC-pLKO.1-puro from Sigma (St. Louis, MO, USA). The CerS6 shRNA and NC shRNA sequences have previously been published [13]. K562 and HL-60 cells (1 × 10^6^) were transfected with 2 µg of CerS6-pLKO.1-puro or NC-pLKO.1-puro using Lipofectamine 2000 (Invitrogen, Carlsbad, CA, USA), following the manufacturer’s recommended protocol. Starting at 24 h post-transfection, the cells were selected with 2 µg/mL puromycin for 14 days to obtain stable clones, and positive clones were picked for identification. Stable cell lines were cultured in RPMI1640 supplemented with 10% FBS, 2 μg/mL puromycin, 100 U/mL penicillin, and 100 μg/mL streptomycin (Gibco). Cultures were maintained at 37°C in a humidified atmosphere of 95% air/5% CO_2_.

### Plasmids and transfection

K562 and HL-60 cells (1 × 10^6^) were transfected with 6 μg of DN-JNK expression vector or empty vector (pUSEamp) (Upstate Technology, Lake Placid, NY, USA) using Lipofectamine following the manufacturer’s protocol. After transfection, cells were cultured for 24 h in RPMI-1640 supplemented with 10% FBS, and then treated for 6 h with sterilized water or cladoloside C_2_. These cells were analyzed by annexin-V staining, immunofluorescence, and western blot.

### Establishment of HL-60 and K562 leukemia xenograft models

All animal procedures and care were approved by the Institutional Animal Care and Usage Committee of Dong-A University. To determine the *in vivo* activity of cladoloside C_2_, K562 cells (1.5 × 10^7^/100 μL PBS per mouse) and HL-60 cells (2 × 10^7^/100 μL PBS per mouse), which were confirmed to be viable by trypan blue staining, were injected into the right flanks of 6-week-old female Balb/c nude mice (*n* = 5 mice per group; Orient Bio Inc., Korea), as previously described [12, 29]. To confirm the essential role of CerS6 in the *in vivo* antitumor activity of cladoloside C_2_, K562 and HL-60 cells expressing NC construct and K562 and HL-60 cells expressing CerS6 shRNA were injected, at the above-mentioned concentrations, into the right flanks of 6-week-old female Balb/c nude mice (*n* = 5 mice per group; Orient Bio Inc., Korea). When the average subcutaneous tumor volume reached 60–100 mm^3^, the mice were assigned to either the cladoloside C_2_ treatment or control group, receiving 7.19 μg/kg cladoloside C_2_ or vehicle via the tail vein every 3 days. Tumor size was measured using a caliper: calculated volume = shortest diameter^2^ × longest diameter/2. Mice were followed for tumor size and body weight, and were sacrificed on the 14^th^ or 21^st^ day. Tumors were resected, weighed, and frozen or fixed in formalin and paraffin embedded for western blot or immunohistochemical analyses.

### Histology and immunohistochemical analysis

Tumor sections were stained with hematoxylin/eosin, and immunohistochemistry was performed using the Discovery XT automated immunohistochemistry stainer (Ventana Medical Systems, Inc., Tucson, AZ, USA). Tissue sections were deparaffinized using EZ Prep solution (Ventana Medical Systems). Antigen retrieval was performed by using CCl standard (pH 8.4 buffer containing Tris/Borate/EDTA; Ventana Medical Systems) for 24 min with anti-Fas antibody, 45 min with anti-ceramide antibody, 24 min with anti-CerS6 antibody, 45 min with anti-p-p38 antibody, 60 min with anti-p38 antibody, 15 min with anti-p-JNK antibody, and 24 min with anti-JNK antibody. The slides were then treated with Inhibitor D (3% H_2_O_2_, endogenous peroxidase; Ventana Medical Systems) for 4 min at 37°C. Next, the slides were incubated at 37°C for 32 min with anti-Fas antibody (1:50 dilution; Santa Cruz Biotechnology, Santa Cruz, CA, USA), 1 h with anti-ceramide antibody (1:10 dilution; Enzo Life Sciences, Inc., PA, USA), 30 min with anti-CerS6 antibody (1:200 dilution; Biorbyt Limited, Cambridge, UK), 1 h with anti-p-p38 antibody (1:200 dilution; Cell Signaling Technology, Beverly, MA, USA), 2 h with anti-p38 antibody (1:20 dilution; Developmental Studies Hybridoma Bank, Iowa City, IA, USA), and 32 min with anti-JNK antibody (1:20 dilution; Cell Signaling Technology, Beverly, MA, USA). The slides were then incubated overnight at 4°C with anti-p-JNK antibody (1:20 dilution; Cell Signaling Technology, Beverly, MA, USA). Subsequently, the slides were treated at 37°C for 8 min with Dako REAL™ Envision™ anti-rabbit/mouse HRP (Dako) secondary antibody to anti-ceramide antibody, anti-CerS6 antibody, and anti-p-p38 antibody; for 16 min with the secondary antibody to anti-Fas antibody, anti-p38 antibody, and anti-JNK antibody; and for 30 min with the secondary antibody to anti-p-JNK antibody. Finally, the slides were incubated for 8 min in DAB^+^ H_2_O_2_ substrate using the Ventana Chromo Map Kit (Ventana Medical Systems), followed by hematoxylin/eosin counterstaining. Sections were washed with PBS, mounted with VectaShield mounting medium (Vector Laboratories, Burlingame, CA, USA), coverslipped, and imaged using a ScanScope (Aperio Technologies, Inc., Vista, CA, USA).

### Statistical analysis

Statistical analyses were performed using the SPSS 21.0 statistical package for Windows (SPSS, Chicago, IL, USA). Data are expressed as mean ± standard deviation (SD). We used one-way ANOVA to evaluate significant differences in cell viability between cladoloside C_2_-treated and control cells. We assessed differences in tumor volume between treated and control groups using Student’s unpaired *t*-test. Statistical significance was defined as *P* < 0.05.

## SUPPLEMENTARY MATERIALS FIGURES



## Abbreviations

STC: Sticholoside C
STD: Stichoposide D
CerS6: Ceramide synthase 6
SMases: Sphingomyelinases
PARP: Poly (ADP-ribose) polymerase
JNK: c-Jun-NH_2_-terminal kinase
FBS: Fetal bovine serum
MMP: Mitochondrial membrane potential
DN-JNK: Dominant negative- JNK
Mcl-1: Myeloid cell leukemia-1 (Mcl-1)
Bcl-2: B-cell lymphoma-2
Bcl-xL: B-cell lymphoma extra large
MAPKs: Mitogen-activated protein kinases
ERK: Extracellular signal regulating kinase
MβCD: Methyl-β-cyclodextrin
CTx: cholera toxin
