# Supplementary material for: In vitro and in vivo anti-leukemic effects of cladoloside C2 are mediated by activation of Fas/ceramide synthase 6/p38 kinase/c-Jun NH2-terminal kinase/caspase-8
Source: Oncotarget. 2017 Dec 8;9(1):495–511. doi: 10.18632/oncotarget.23069 (PMC5787484; doi:10.18632/oncotarget.23069)
Supplement: Supplementary file 1 [file oncotarget-09-495-s001.pdf]

In vitro and *in vivo* anti-leukemic effects of cladoloside C_2 are mediated by activation of Fas/ceramide synthase 6/p38 kinase/c-Jun NH_2-terminal kinase/caspase-8

SUPPLEMENTARY MATERIALS

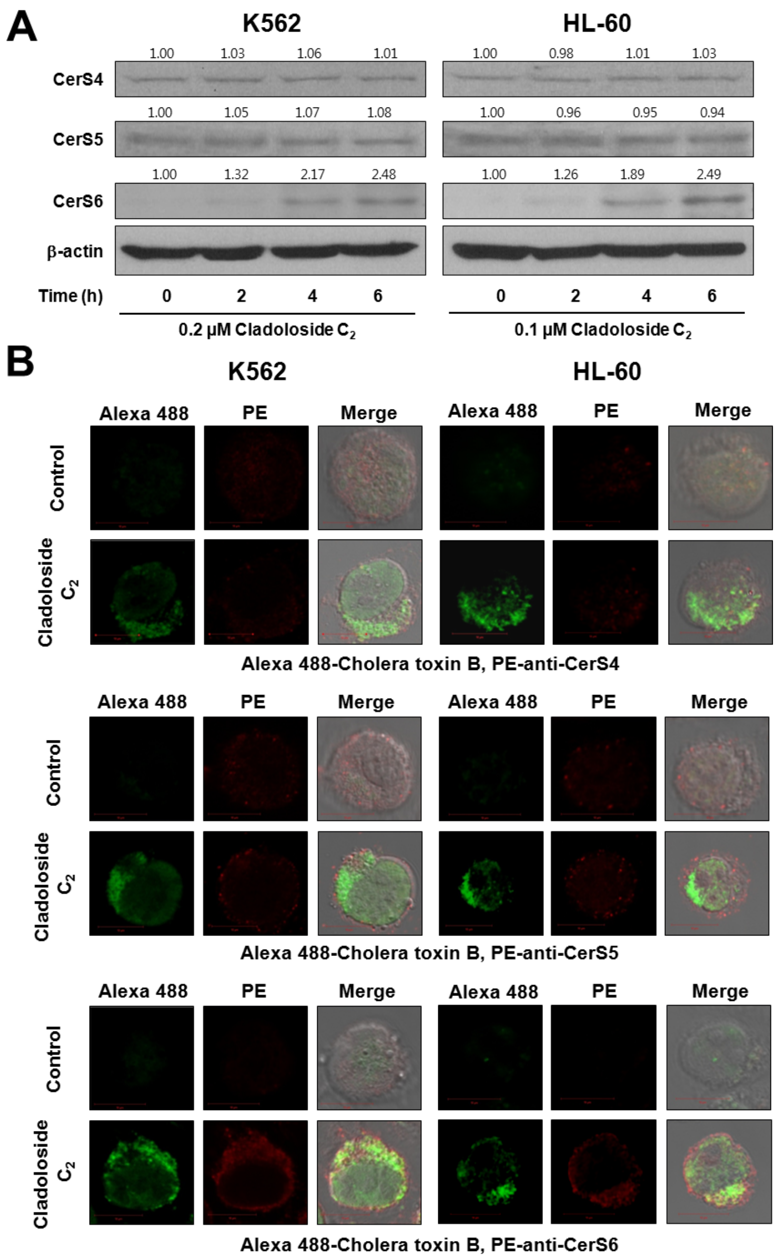

Supplementary Figure 1: Cladoloside C_2 induces the expression of CerS6, but not CerS4 or CerS5, in K562 and HL-60 cells. K562 and HL-60 cells were treated with cladoloside C_2 for the indicated times. (**A**) Protein lysates were prepared and subjected to western blot analysis. β-actin was used as a loading control. The blot is representative of three separate experiments. (**B**) After permeabilization, samples were stained with PE-anti-CerS4, PE-anti-CerS5, or PE-anti-CerS6 antibodies and Alexa 488-labeled cholera toxin B antibody. The pictures are representative of three separate experiments.

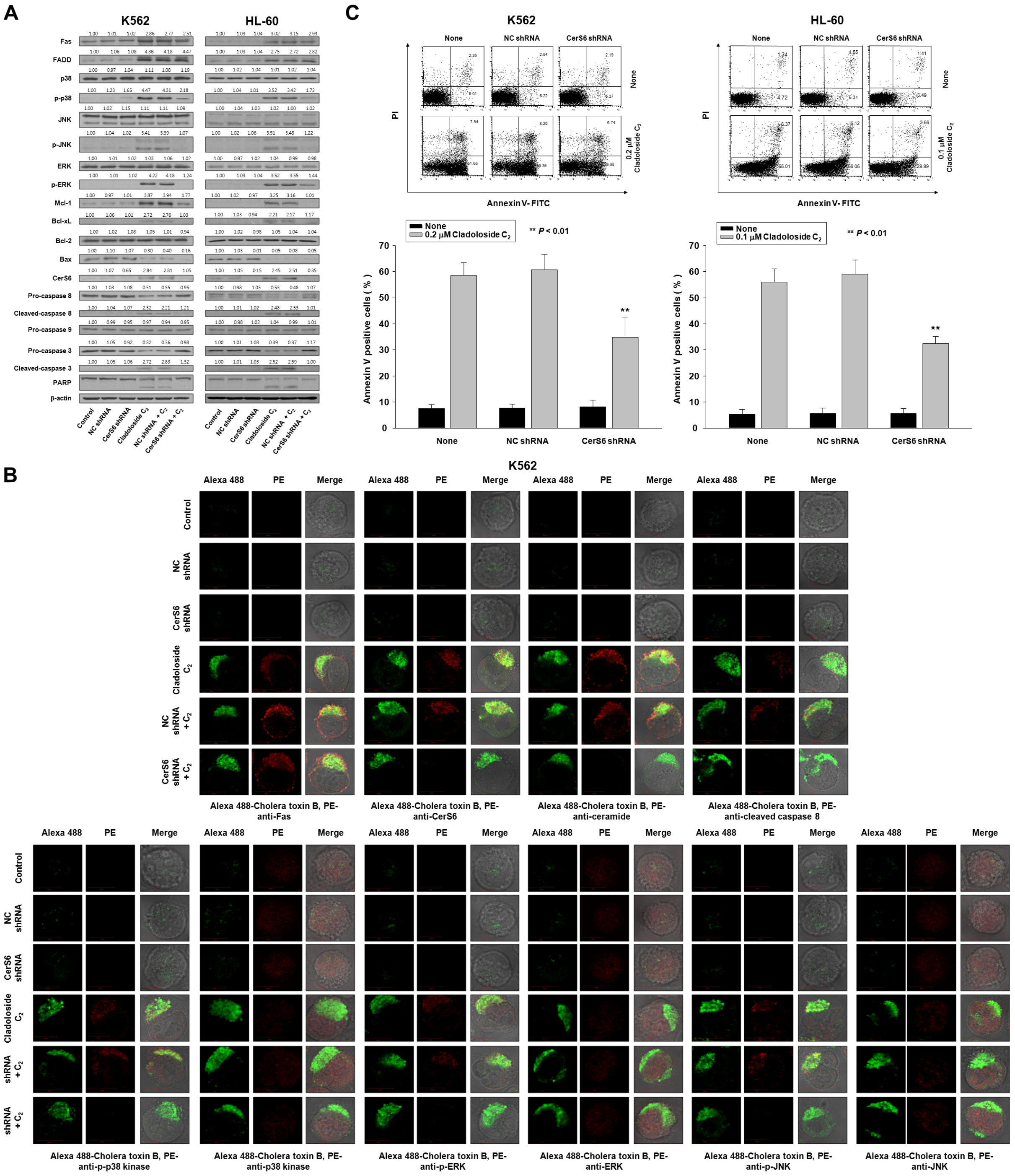

Supplementary Figure 2: Cladoloside C_2 induces apoptosis of K562 and HL-60 cells through the activation of ceramide synthase 6 (CerS6). (**A–C**) Control, NC-shRNA-transfected, and CerS6 shRNA-transfected stable K562 and HL-60 cells were incubated for 6 h with or without cladoloside C_2. (A) Western blot analysis of protein lysates. (B) Control, NC-shRNA-transfected, and CerS6 shRNA-transfected stable K562 cells were exposed to 0.2 μM cladoloside C_2 for 2 h, and then fixed and permeabilized. Samples were then stained with PE-conjugated antibodies against Fas, CerS6, ceramide, cleaved caspase-8, p-p38 kinase, p38 kinase, p-ERK, ERK, p-JNK, or JNK, and with Alexa 488-labeled cholera toxin B antibody. The pictures are representative of three separate experiments. (C) Upper panel: The culture medium was changed, and K562 and HL-60 cells were incubated for 6 h with or without 0.2 or 0.1 μM cladoloside C_2. The percentage of apoptotic cells was determined by annexin V-FITC/PI staining. Results are representative of three independent experiments in each cell line. Lower panel: Mean ± SD of three independent experiments. $^{**}P < 0.01$, $^{***}P < 0.001$, cells treated with cladoloside C_2 alone versus CerS6 shRNA-silenced cells treated with cladoloside C_2.

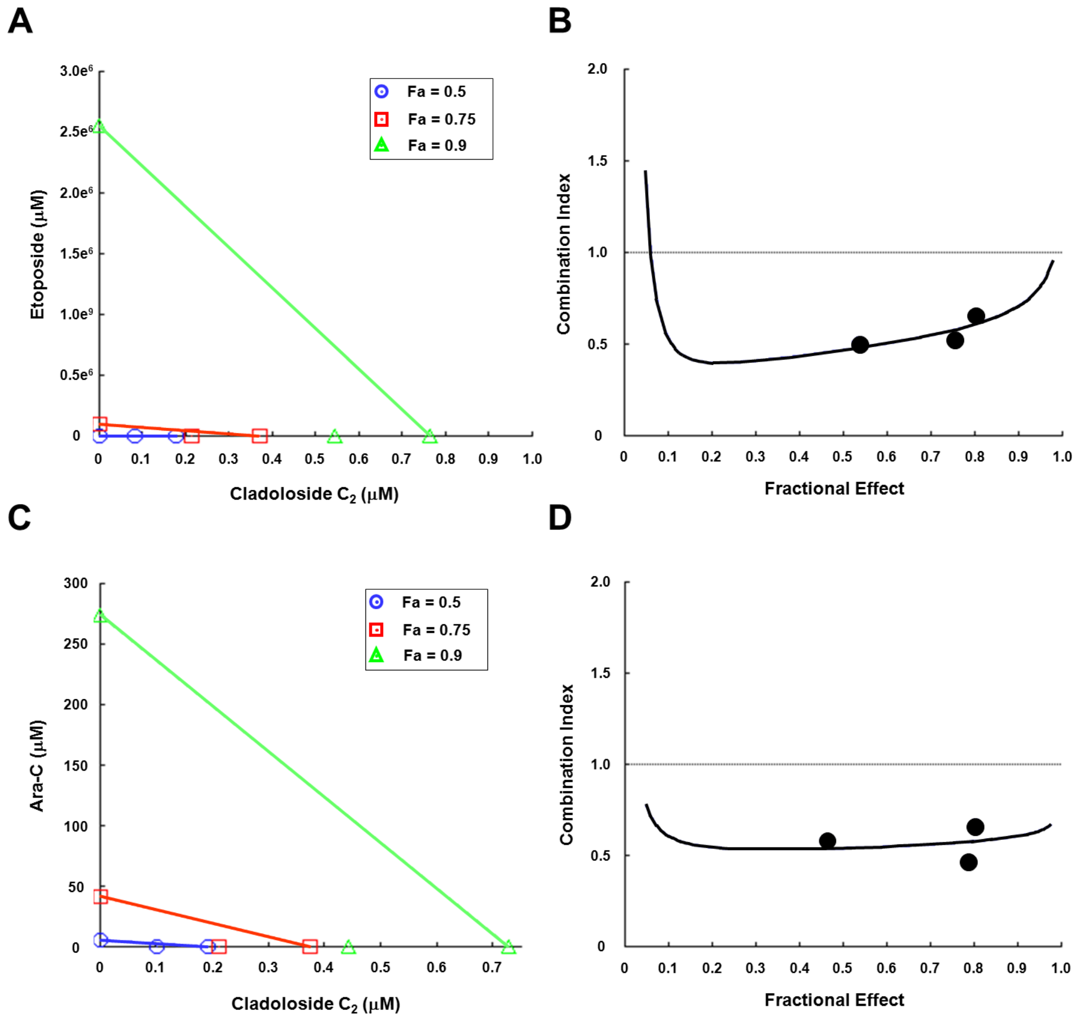

Supplementary Figure 3: Cladoloside C_2 enhances the chemosensitivity of K562 cells to etoposide or Ara-C. (**A**, **C**) Isobolograms of the combination of cladoloside C_2 with etoposide (A) or Ara-C (C). (**B**, **D**) Combination index (CI) of cladoloside C_2 with etoposide (B) or Ara-C (D). CI was determined by CalcuSyn software version 2.0 (Biosoft, Cambridge, UK).
